# Supplementary material for: Digital health interventions for gestational diabetes mellitus: A systematic review and meta-analysis of randomised controlled trials
Source: PLOS Digit Health. 2022 Feb 24;1(2):e0000015. doi: 10.1371/journal.pdig.0000015 (PMC9931335; doi:10.1371/journal.pdig.0000015)
Supplement: S1 Appendix — (DOCX) [file pdig.0000015.s001.docx]

# **Digital health interventions for gestational diabetes mellitus: A systematic review and meta-analysis of randomised controlled trials on maternal and neonatal outcomes**

**Boutheina Leblalta, Hanane Kebaili, Ruth Sim, Shaun Wen Huey Lee**

Box A: Search strategy used in this study for each database

| **Database searched** | **Search terms** |
| --- | --- |
| Ovid MEDLINE, Cochrane Central Register of Controlled Trials (CENTRAL), Embase, CINAHL Plus, PsychINFO, Maternal & Infant Care Database (MIDIRS) | (Gestational diabetes mellitus or GDM or gestational diabetes or diabetes, pregnancy induced or diabetes mellitus, gestational or pregnancy, glucose intolerance or pregnancy, hyperglyc*emia or pregnancy glyc*emic index OR pregnancy-induced diabetes or diabetes, gestational) AND (telehealth or digital health or telemonitoring or telecare or telematics or telehomecare or mobile health or remote monitoring or remote consultation or sms or short messaging system or video monitoring or internet monitoring or internet consultation or video consultation or telephone or smartphone or cellular phone or mobile phone or telephone support) AND (randomi*ed controlled trial or controlled clinical trial) |
| CNKI database | 妊娠糖尿病 AND 远程医疗 |
| ClinicalTrials.gov, WHO Clinical Trial Registry | Gestational diabetes AND digital health OR internet |

Fig A: Detailed risk of bias for each study included in the current review

|  | Randomization process | Deviations from intended interventions | Missing outcome data | Measurement of the outcome | Selection of the reported result | **Overall risk of bias** |
| --- | --- | --- | --- | --- | --- | --- |
| Al-Ofi 2019 |  |  |  |  |  |  |
| Bartholomew 2015 |  |  |  |  | |  |
| Borgen 2019 |  |  |  |  | |  |
| Bromuri 2016 |  |  |  |  | |  |
| Caballero Ruiz 2017 |  |  |  |  | |  |
| Carolan-Olah 2019 |  |  |  |  | |  |
| Cui 2019 |  |  |  |  | |  |
| Given 2015 |  |  |  |  | |  |
| Guo 2019 |  |  |  |  | |  |
| Homko 2007 |  |  |  |  | |  |
| Homko 2012 |  |  |  |  | |  |
| Huang 2020 |  |  |  |  | |  |
| Huang 2021 |  |  |  |  | |  |
| Jiang 2016 |  |  |  |  | |  |
| Jiang 2018 |  |  |  |  | |  |
| Khorshidi Roozbahani 2015 |  |  |  |  | |  |
| Liu 2018 |  |  |  |  | |  |
| Mackillop 2018 |  |  |  |  | |  |
| Miremberg 2018 |  |  |  |  | |  |
| Pérez-Ferre 2010 |  |  |  |  | |  |
| Rasekaba 2018 |  |  |  |  | |  |
| Sung 2019 |  |  |  |  | |  |
| Tian 2021 |  |  |  |  | |  |
| Weng 2018 |  |  |  |  | |  |
| Yew 2021 |  |  |  |  | |  |
| Yu 2019 |  |  |  |  | |  |
| Zhao 2018 |  |  |  |  | |  |
| Zeng 2017 |  |  |  |  | |  |

**Legend**

|  | Low risk |
| --- | --- |
|  | Some concerns |
|  | High risk |

Fig B: Forest plot for weight gain during pregnancy for digital health interventions compared with routine care in women with GDM. Reference citations for studies can be found in Table 1.


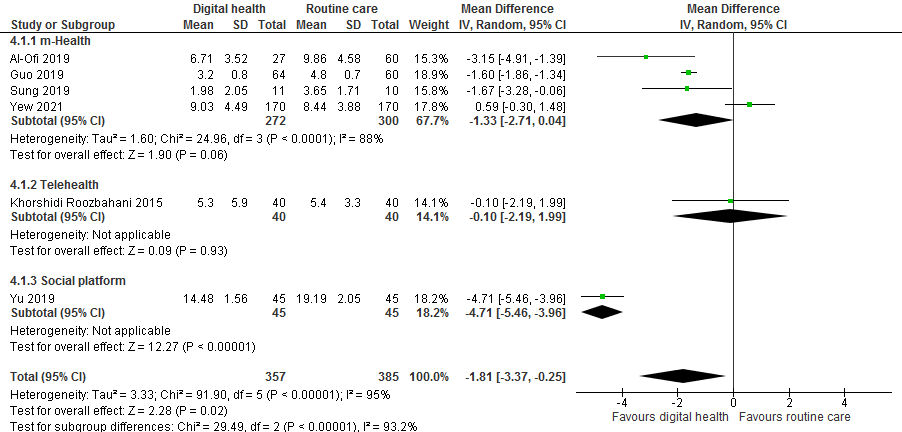


Fig C: Forest plot comparing the need for caesarean section for digital health interventions compared to routine care in women with GDM. Reference citations for studies can be found in Table 1.


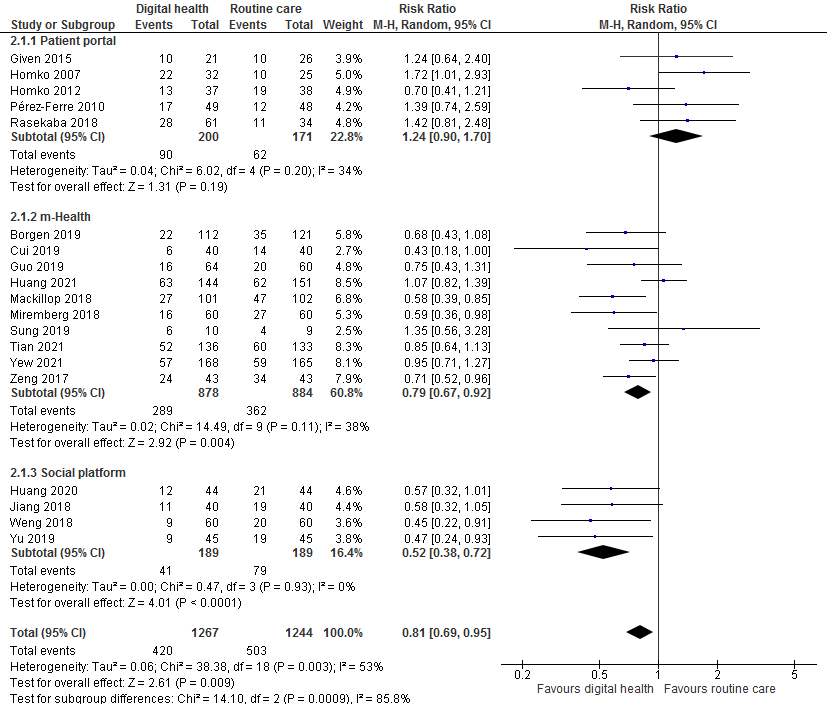


Fig D: Forest plot on the incidence of pre-eclampsia / eclampsia for digital health interventions compared with routine care in women with GDM. Reference citations for studies can be found in Table 1.


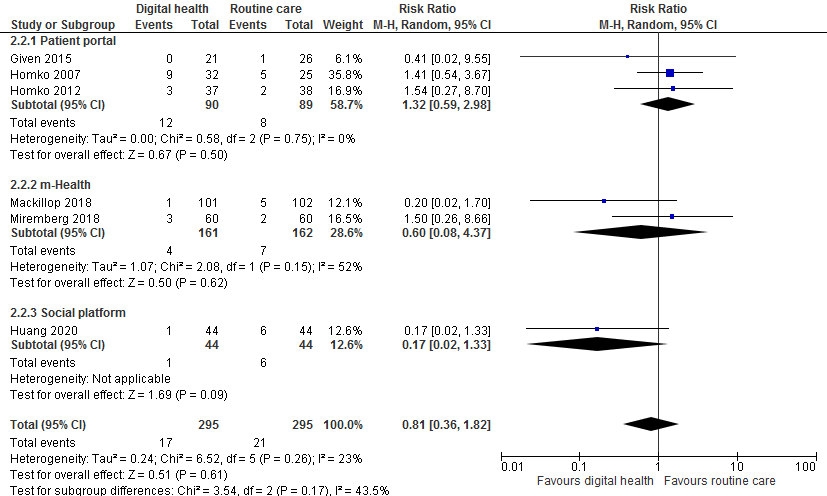


Fig E: Forest plot of medication use during pregnancy for digital health interventions compared with routine care in women with GDM. Reference citations for studies can be found in Table 1.


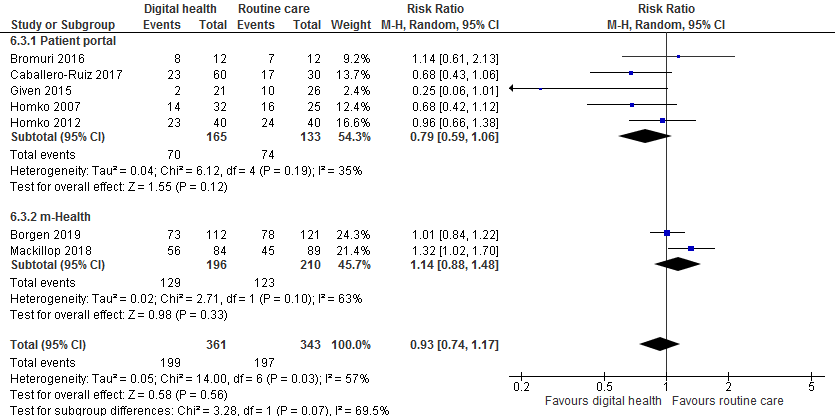


Fig F: Forest plot for the need of neonatal intensive care unit use for digital health interventions compared with routine care in women with GDM. Reference citations for studies can be found in Table 1.


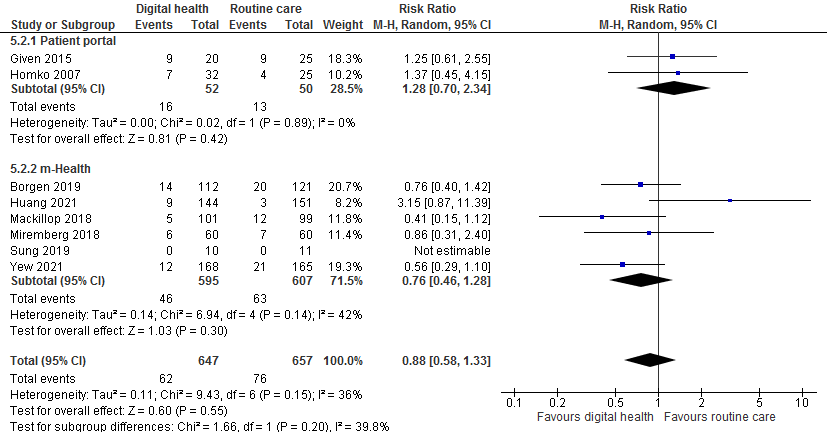


Fig G: Forest plot of incidence for preterm birth for digital health interventions compared with routine care in women with GDM. Reference citations for studies can be found in Table 1.


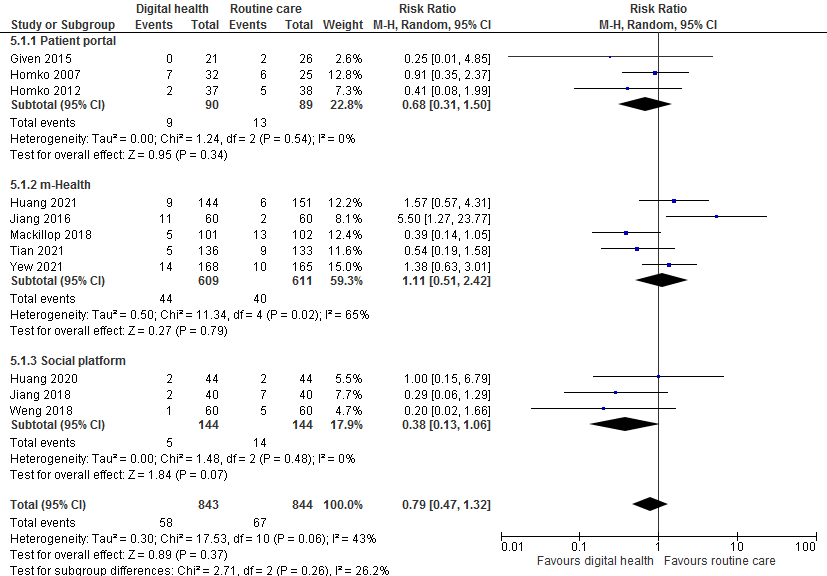


Fig H: Forest plot on incidence of foetal macrosomia for digital health interventions compared with routine care in women with GDM. Reference citations for studies can be found in Table 1.


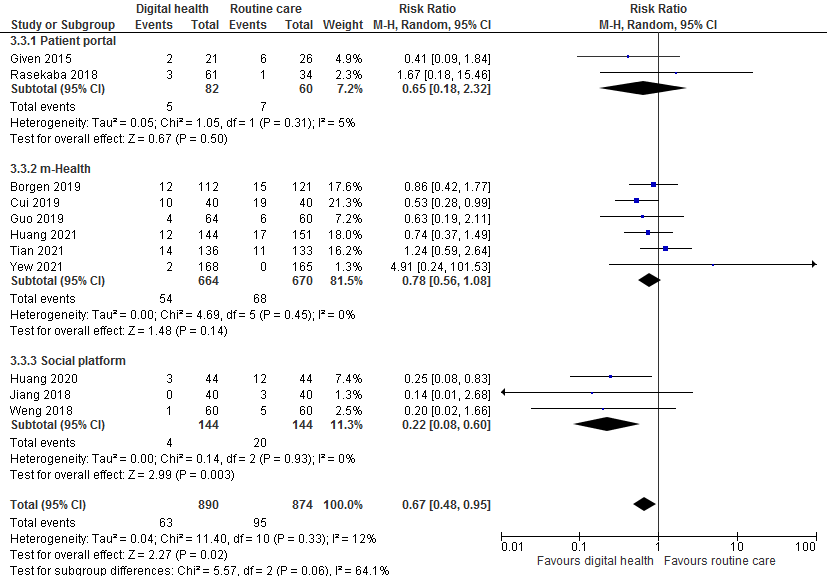


Fig I: Forest plot of incidence for large for gestational age for digital health interventions compared with routine care in women with GDM. Reference citations for studies can be found in Table 1.


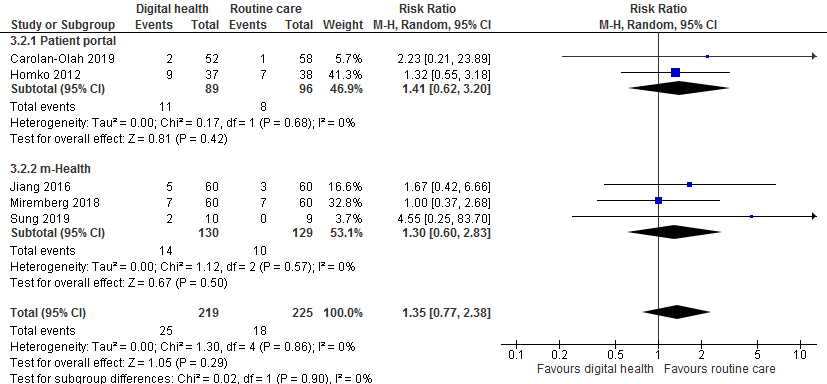


Fig J: Forest plot of infant birth weight for digital health interventions compared with routine care in women with GDM. Reference citations for studies can be found in Table 1.


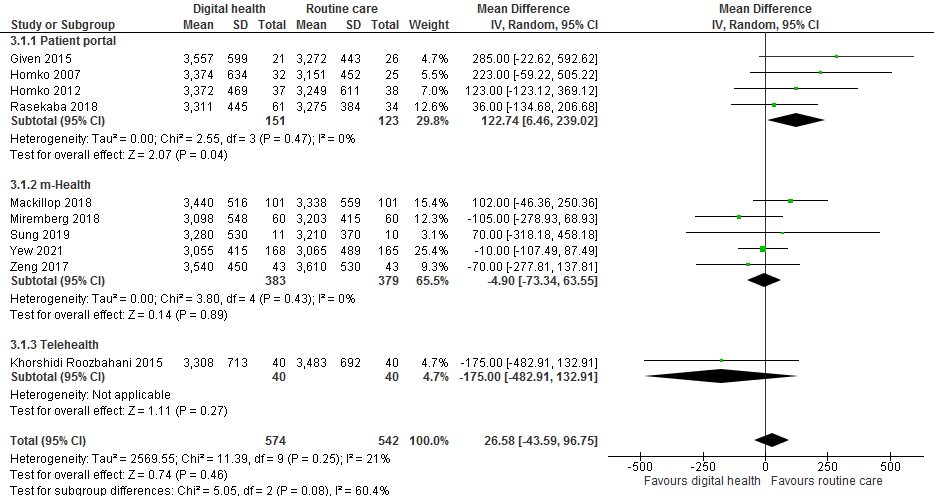


Fig K: Forest plot of incidence of hypoglycaemia among new born for digital health interventions compared with routine care in women with GDM. Reference citations for studies can be found in Table 1.


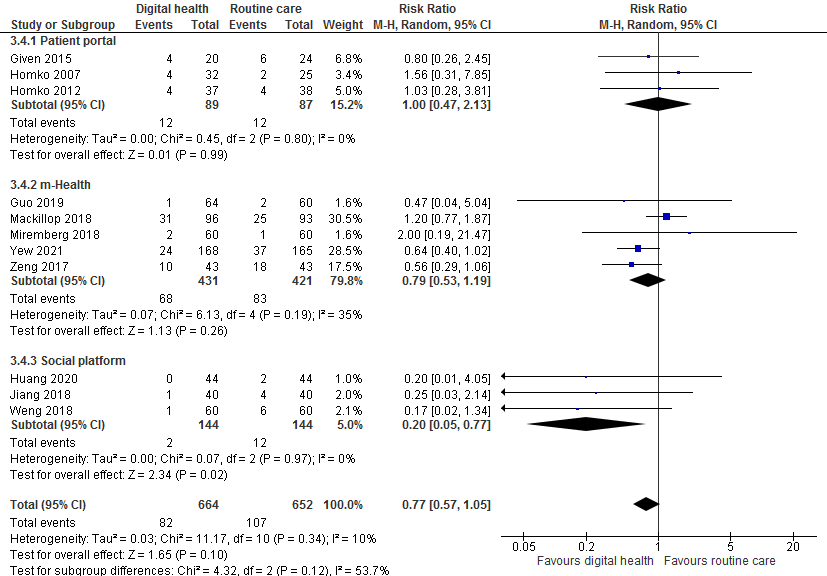


Fig L: Forest plot of incidence for small for gestational age for digital health interventions compared with routine care in women with GDM. Reference citations for studies can be found in Table 1.


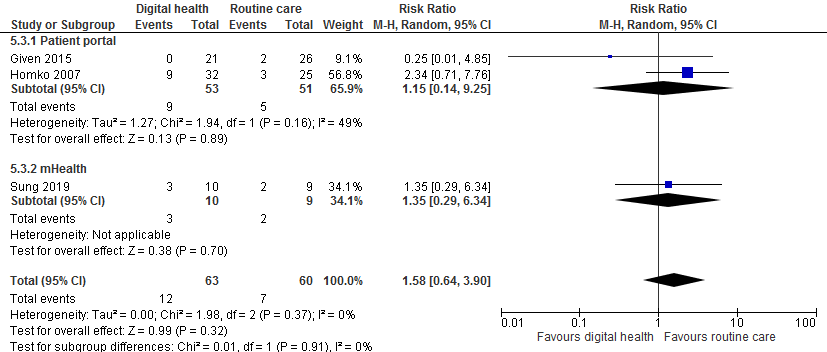


Fig M: Funnel plots for all maternal outcomes

| 1. Fasting plasma glucose | 1. 2-hour post prandial glucose |
| --- | --- |
| **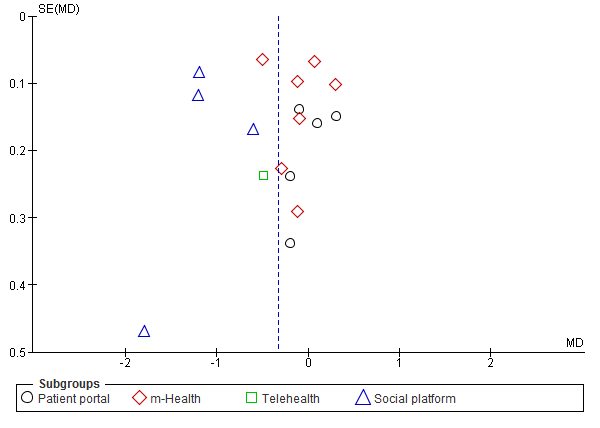** | **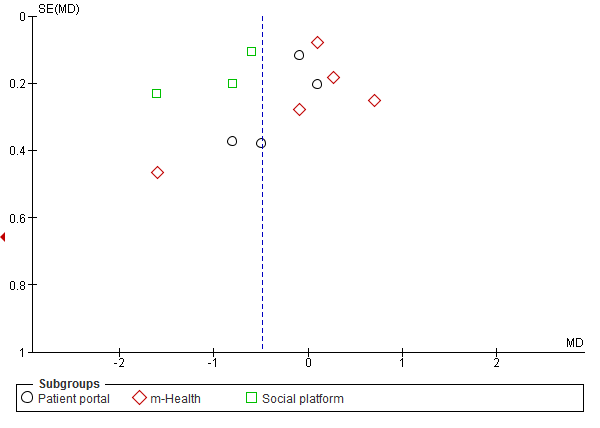** |
|  |  |
| 1. HbA_1c_ | 1. Weight gain over pregnancy |
|  |  |
| **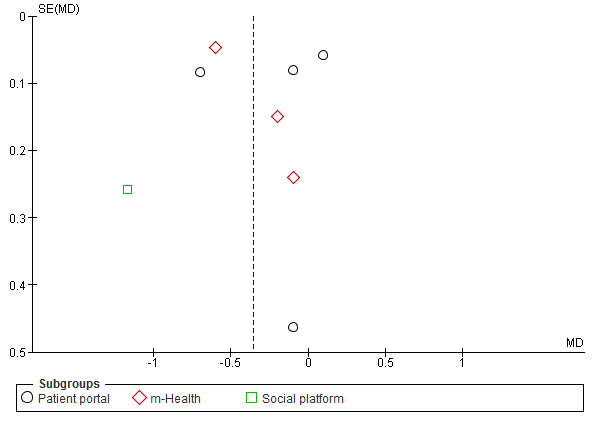** | **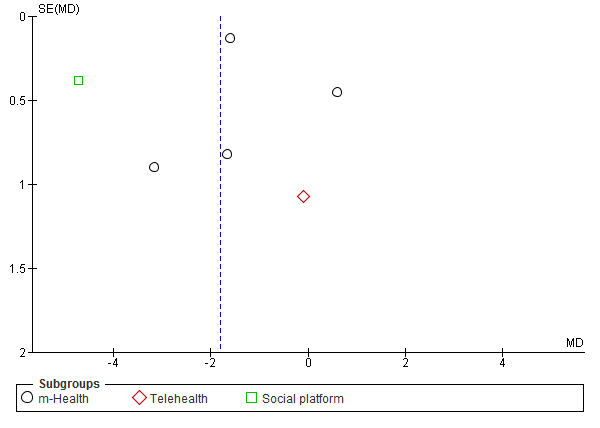** |
|  |  |
| 1. Medication use during pregnancy | 1. Caesarean section |
| **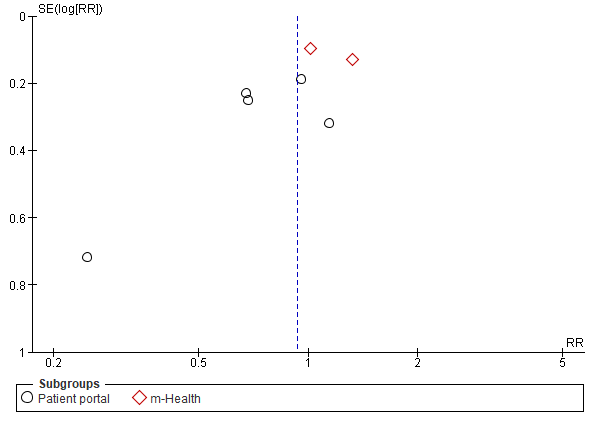** | **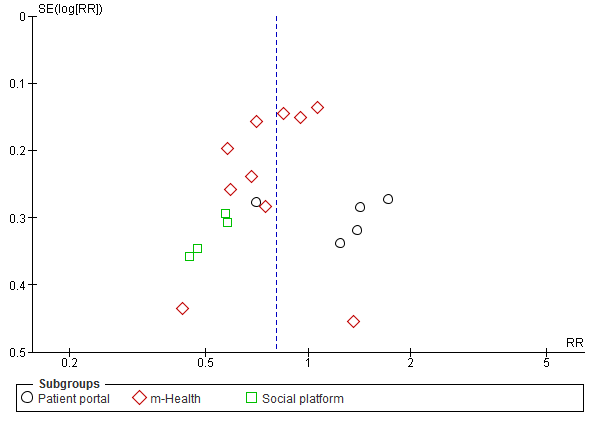** |
|  |  |
| 1. Incidence of pre-eclampsia/eclampsia |  |
| **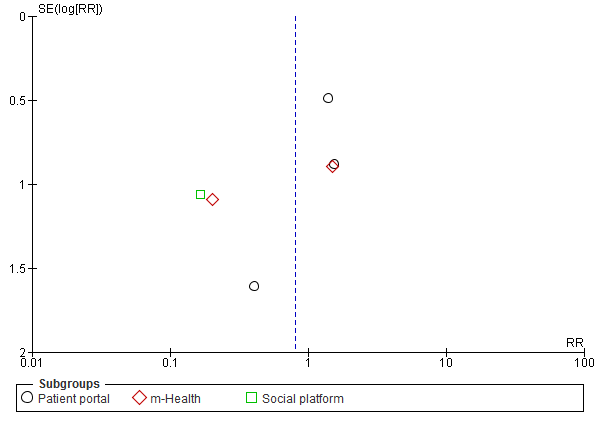** |  |

Fig N: Funnel plots for all neonatal outcomes

| 1. Infant birth weight | 1. Incidence of foetal macrosomia |
| --- | --- |
| 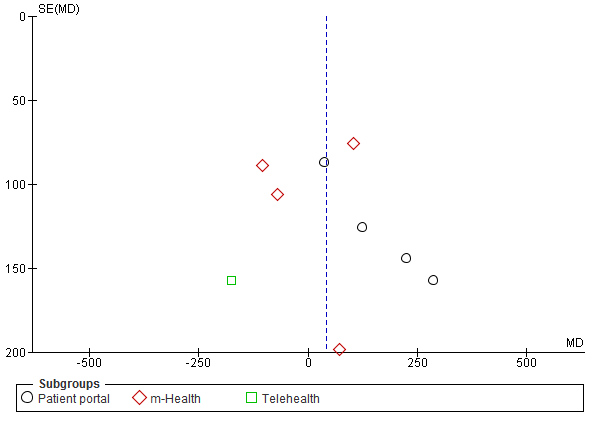 | 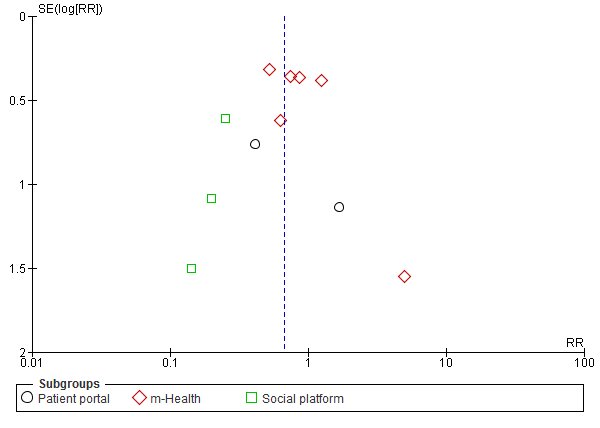 |
|  |  |
| 1. Incidence of large for gestational age | 1. Incidence of small for gestational age |
| 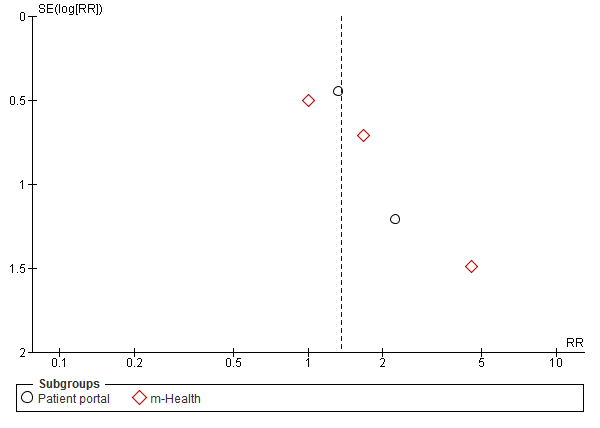 | 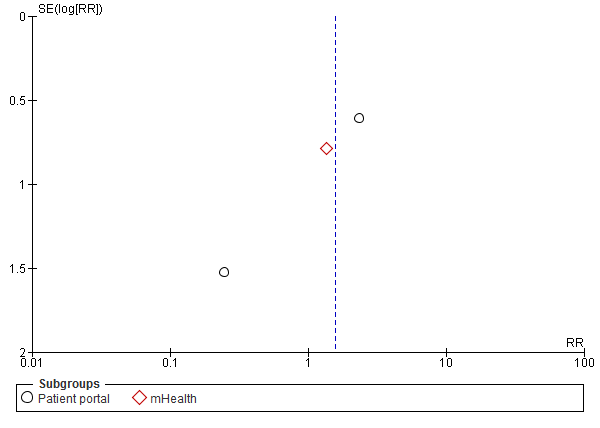 |
|  |  |
| 1. Incidence of hypoglycaemia of newborn | 1. Preterm birth |
| 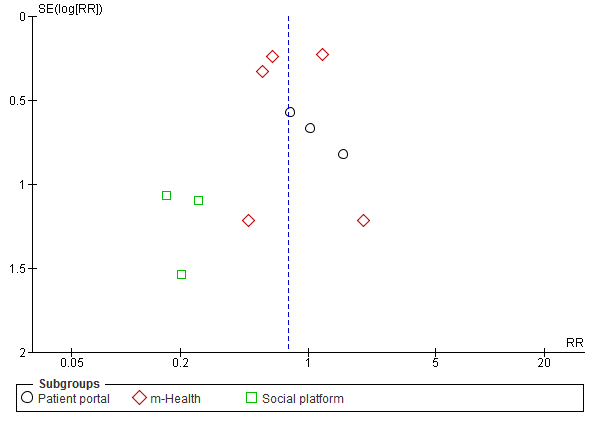 | 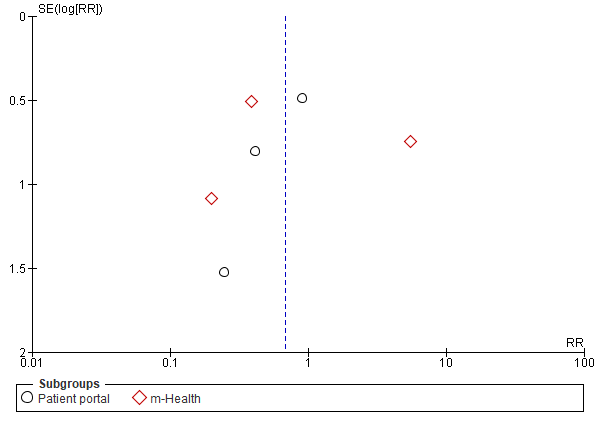 |
| 1. Neonatal intensive care unit |  |
| 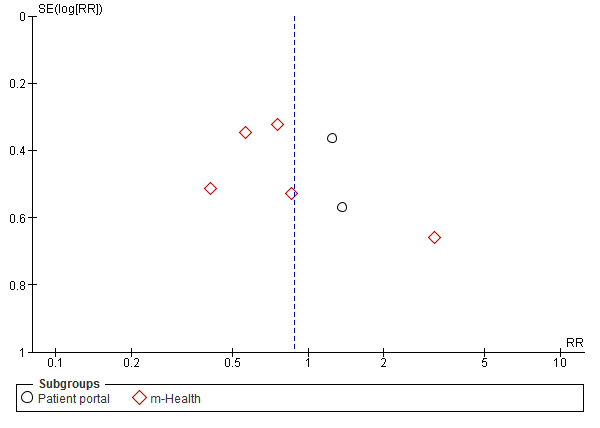 |  |

Table A: Definition of study compliance as reported in each trial

| **Author, Year (Country)** | **Definition of study compliance as reported in study** |
| --- | --- |
| Cui et al^36^, 2019 (China) | Number of participants who followed recommendations of trial |
| Guo et al^17^,2019 | Number of blood glucose measurements performed compared to number of blood glucose measurements instructed |
| Jiang et al^38^, 2018 (China) | Number of participants who followed recommendations of trial |
| Miremberg et al^28^, 2018 | Number of blood glucose measurements performed compared to number of blood glucose measurements instructed |
| Mackillop et al^41^, 2018 | Number of participants who recorded/reported at least 67% of the recommended blood glucose measurements |
| Homko et al^48^, 2007 | Total number of blood glucose measurements performed during the study period |

Table B: Subgroup analysis on glycaemic outcomes

| **Outcome** | ***N* of studies** | ***N* of women** | **Mean effect estimate [95% CI]** | ***I*^2^ (%)** |
| --- | --- | --- | --- | --- |
| **Change in fasting plasma glucose (mmol/L)** |  |  |  |  |
| All comparison | 17 | 1,529 | -0.33 [-0.59, -0.07] | 94 |
| Intervention classification |  |  |  |  |
| Telemonitoring | 12 | 1,145 | -0.08 [-0.27, 0.10] | 86 |
| Tele-education | 5 | 464 | -0.90 [-1.46, -0.34] | 95 |
| Study sample size |  |  |  |  |
| <100 participants | 13 | 896 | -0.30 [-0.65, 0.05] | 95 |
| >100 participants | 4 | 633 | -0.39 [-0.82, 0.04] | 94 |
| Study location |  |  |  |  |
| Asia | 14 | 1,373 | -0.39 [-0.69, -0.09] | 95 |
| Europe | 1 | 24 | -0.20 [-0.67, 0.27] | - |
| North America | 2 | 132 | -0.01 [-0.22, 0.19] | 0 |
| GDM diagnosis criteria |  |  |  |  |
| Not reported / Others | 6 | 532 | -0.68 [-1.10, -0.27] | 91 |
| IADPSG criteria | 5 | 493 | 0.01 [-0.17, 0.18] | 53 |
| 2011 ADA criteria | 4 | 372 | -0.40 [-1.13, 0.33] | 97 |
| Carpenter and Coustan | 2 | 132 | -0.01 [-0.22, 0.19] | 0 |
| **Change in postprandial glucose (mmol/L)** |  |  |  |  |
| All comparison | 13 | 1,356 | -0.49 [-0.83, -0.15] | 95 |
| Intervention classification |  |  |  |  |
| Telemonitoring | 10 | 1,058 | -0.17 [-0.45, 0.11] | 81 |
| Teleeducation | 3 | 298 | -1.68 [-2.80, -0.57] | 94 |
| Study sample size |  |  |  |  |
| <100 participants | 9 | 685 | -0.58 [-0.95, -0.21] | 91 |
| >100 participants | 4 | 671 | -0.41 [-1.36, 0.54] | 91 |
| Study location |  |  |  |  |
| Asia | 10 | 1,066 | -0.76 [-1.20, -0.31] | 94 |
| Europe | 1 | 158 | 0.70 [0.21, 1.19] | - |
| North America | 2 | 132 | -0.05 [-0.25, 0.15] | 0 |
| GDM diagnosis criteria |  |  |  |  |
| Not reported / Others | 4 | 466 | -1.13 [-2.60, 0.35] | 95 |
| IADPSG criteria | 3 | 386 | -0.65 [-1.77, 0.46] | 89 |
| 2011 ADA criteria | 4 | 372 | -0.35 [-0.83, 0.13] | 92 |
| Carpenter and Coustan | 2 | 132 | -0.05 [-0.25, 0.15] | 0 |
| **Change in HbA1c levels (%)** |  |  |  |  |
| All comparison | 8 | 621 | -0.36 [-0.65, -0.07] | 96 |
| Intervention classification |  |  |  |  |
| Telemonitoring | 7 | 501 | -0.26 [-0.57, 0.04] | 95 |
| Teleeducation | 1 | 120 | -1.17 [-1.67, -0.67] | - |
| Study sample size |  |  |  |  |
| <100 participants | 6 | 377 | -0.20 [-0.51, 0.12] | 92 |
| >100 participants | 2 | 244 | -0.83 [-1.38, -0.28] | 79 |
| Study location |  |  |  |  |
| Asia | 5 | 420 | -0.55 [-0.77, -0.33] | 78 |
| Europe | 2 | 144 | 0.01 [-0.19, 0.20] | 75 |
| North America | 1 | 57 | -0.10 [-1.01, 0.81] | - |
| GDM diagnosis criteria |  |  |  |  |
| Not reported / Others | 3 | 265 | -0.56 [-1.25, 0.13] | 97 |
| IADPSG criteria | 2 | 78 | -0.17 [-0.42, 0.08] | 0 |
| 2011 ADA criteria | 1 | 124 | -0.60 [-0.69, -0.51] | - |
| Carpenter and Coustan | 2 | 154 | -0.10 [-0.26, 0.06] | 0 |

ADA: American Diabetes Association; IADPSG- International Association of Diabetes and Pregnancy Study Group

Table C: Pooled analyses of maternal and neonatal outcomes after inclusion of high quality studies only

| **Outcome** | ***N* of studies** | ***N* of women** | **Effect estimate** | ***I*^2^ (%)** |
| --- | --- | --- | --- | --- |
| **Maternal outcomes** |  |  |  |  |
|  |  |  | **Mean difference [95% CI]** |  |
| Change in fasting glucose (mmol/L) | 3 | 224 | -0.48 [-0.60, -0.36] | 0 |
| Change in postprandial glucose (mmol/L) | 1 | 158 | 0.70 [0.21, 1.19] | - |
| Change in HbA_1c_ (%) | 1 | 47 | 0.10 [-0.02, 0.22] | - |
| Weight gain over pregnancy (kg) | 2 | 420 | 0.48 [-0.34, 1.30] | 0 |
| Infant birth weight (g) | 6 | 877 | 14.31 [-74.85, 103.48] | 36 |
|  |  |  | **RR [95% CI]** |  |
| Caesarean delivery rates | 6 | 1,031 | 0.83 [0.62, 1.10] | 57 |
| Incidence of preeclampsia/ eclampsia | 3 | 370 | 0.61 [0.17, 2.23] | 7 |
| Use of medication | 4 | 507 | 0.78 [0.52, 1.17] | 0 |
| Hypoglycaemia of new-born | 4 | 686 | 0.89 [0.59, 1.35] | 29 |
| Preterm birth | 3 | 583 | 0.68 [0.23, 1.97] | 55 |
| Neonatal intensive care unit | 5 | 931 | 0.74 [0.52, 1.06] | 6 |
| Incidence of foetal macrosomia | 4 | 708 | 0.86 [0.47, 1.58] | 0 |
| Large for gestational age | 1 | 120 | 1.00 [0.37, 2.68] | - |
| Small for gestational age | 1 | 47 | 0.25 [0.01, 4.85] | - |

#### Table D: GRADE summary of findings on maternal outcomes with use of digital health interventions in gestational diabetes

| Outcomes | **Anticipated absolute effects^*^** (95% CI) | | Relative effect (95% CI) | № of participants  (studies) | Certainty of the evidence (GRADE) | **Comments** |
| --- | --- | --- | --- | --- | --- | --- |
|  | **Risk with routine care** | **Risk with digital health** |  |  |  |  |
| Fasting plasma glucose (FPG) |  | MD **0.33 mmol/L lower** (0.59 lower to 0.07 lower) | - | 1529 (17 RCTs) | ⨁⨁⨁◯ MODERATE ^a^ | Use of digital health interventions to supplement routine care probably has some benefit in improving fasting plasma glucose among women with gestational diabetes |
| 2-hr post prandial |  | MD **0.49 mmol/L lower** (0.83 lower to 0.15 lower) | - | 1356 (13 RCTs) | ⨁⨁⨁◯ MODERATE ^a^ | Use of digital health interventions to supplement routine care probably has some benefit in improving 2-hr post prandial glucose among women with gestational diabetes |
| Glycated haemoglobin (HbA1c) |  | MD **0.36 % lower** (0.65 lower to 0.07 lower) | - | 621 (8 RCTs) | ⨁⨁⨁◯ MODERATE ^a^ | Use of digital health interventions to supplement routine care probably has some benefits in improving HbA1c control among women with gestational diabetes |
| Weight gain over pregnancy |  | MD **1.81 kg lower** (3.37 lower to 0.25 lower) | - | 742 (6 RCTs) | ⨁⨁◯◯ LOW ^a,b,c^ | Use of digital health interventions to supplement routine care probably has little to no impact to reduce weight gain during pregnancy among women with gestational diabetes |
| Caesarean | 404 per 1,000 | **328 per 1,000** (279 to 384) | **RR 0.81** (0.66 to 0.95) | 2511 (19 RCTs) | ⨁⨁⨁⨁ HIGH | Use of digital health interventions to supplement routine care probably reduce the need for caesarean delivery among women with gestational diabetes |
| Pre-eclampsia/ Eclampsia | 71 per 1,000 | **58 per 1,000** (25 to 130) | **RR 0.81** (0.35 to 1.82) | 590 (6 RCTs) | ⨁⨁◯◯ LOW ^a,b^ | Use of digital health interventions to supplement routine care probably has little to no impact in reducing the risk of pre-eclampsia/eclampsia among women with gestational diabetes |
| Use of medication | 574 per 1,000 | **534 per 1,000** (425 to 672) | **RR 0.93** (0.74 to 1.17) | 704 (7 RCTs) | ⨁⨁◯◯ LOW ^a,b^ | Use of digital health interventions to supplement routine care probably has little to no impact on the need for medication among women with gestational diabetes |
| ***The risk in the intervention group** (and its 95% confidence interval) is based on the assumed risk in the comparison group and the **relative effect** of the intervention (and its 95% CI).  **CI:** Confidence interval; **MD:** Mean difference; **RR:** Risk ratio | | | | | | |

^a^. Poor description of methodology including method of randomisation, and outcome assessments

^b^. Wide confidence intervals among studies with conflicting evidence

^c^. Unexplained and poor description of outcome

^d^. All studies had small sample sizes

The table describes the classification of the quality of evidence according GRADE guidance

- **Very low quality** which means there is very little confidence in the effect estimate and the effect is likely to be substantially different from the estimate of effect;
- **Low quality** which means there is limited confidence in the effect estimate and the true effect may be substantially different from the estimate of the effect
- **Moderate quality** which means, there is moderate confidence in the effect estimate, and the he true effect is likely to be close to the estimate of the effect, but there is a possibility that it is substantially different;
- **High quality** which there is high confidence that the true effect lies close to that of the estimate of the effect.

#### Table E: GRADE summary of findings on neonatal outcomes with use of digital health interventions in gestational diabetes

| Outcomes | **Anticipated absolute effects^*^** (95% CI) | | Relative effect (95% CI) | № of participants  (studies) | Certainty of the evidence (GRADE) | **Comments** |
| --- | --- | --- | --- | --- | --- | --- |
|  | **Risk with [comparison]** | **Risk with [intervention]** |  |  |  |  |
| Hypoglycaemia of newborn | 164 per 1,000 | **126 per 1,000** (94 to 172) | **RR 0.77** (0.57 to 1.05) | 1316 (11 RCTs) | ⨁⨁⨁◯ MODERATE ^a,b,c^ | Use of digital health interventions to supplement routine care probably has little impact on reducing the risk of hypoglycaemia among newborns |
| Preterm birth | 79 per 1,000 | **63 per 1,000** (37 to 105) | **RR 0.79** (0.47 to 1.32) | 1687 (11 RCTs) | ⨁⨁⨁◯ MODERATE ^a,c,d^ | Use of digital health interventions to supplement routine care probably does not reduce the risk of preterm birth |
| Neonatal intensive care unit | 96 per 1,000 | **84 per 1,000** (56 to 127) | **RR 0.88** (0.58 to 1.33) | 1304 (8 RCTs) | ⨁⨁⨁◯ MODERATE ^a,d^ | Use of digital health interventions to supplement routine care probably does not reduce the need for neonatal intensive care unit hospitalisation among newborns |
| Small for gestational age | 117 per 1,000 | **184 per 1,000** (75 to 455) | **RR 1.58** (0.64 to 3.90) | 123 (3 RCTs) | ⨁◯◯◯ VERY LOW ^c,d^ | Use of digital health interventions to supplement routine care probably has little to no impact on reducing the risk of delivering newborns that are small |
| Foetal macrosomia | 109 per 1,000 | **73 per 1,000** (52 to 103) | **RR 0.67** (0.48 to 0.95) | 1764 (11 RCTs) | ⨁⨁⨁⨁ HIGH | Use of digital health interventions to supplement routine care can reduce the incidence of foetal macrosomia |
| Large for gestational age | 80 per 1,000 | **108 per 1,000** (62 to 190) | **RR 1.35** (0.77 to 2.38) | 444 (5 RCTs) | ⨁◯◯◯ VERY LOW ^a,c,d^ | Use of digital health interventions to supplement routine care probably has little to no impact on reducing the incidence of large for gestational age |
| Infant birth weight |  | MD **26.6 g lower** (43.6 lower to 96.8 higher) | - | 1116 (10 RCTs) | ⨁⨁⨁◯ MODERATE ^a,c^ | Use of digital health interventions to supplement routine care probably has little to no impact on infant birth weight |
| ***The risk in the intervention group** (and its 95% confidence interval) is based on the assumed risk in the comparison group and the **relative effect** of the intervention (and its 95% CI).  **CI:** Confidence interval; **RR:** Risk ratio | | | | | | |

^a^. Poor description of methodology including method of randomisation, blinding and outcome assessments

^b^. Wide confidence intervals among studies

^c.^ Small number of RCTs with small sample sizes

^d^. Limited number of studies reporting outcomes
