# Supplementary material for: Digital health interventions for gestational diabetes mellitus: A systematic review and meta-analysis of randomised controlled trials
Source: PLOS Digit Health. 2022 Feb 24;1(2):e0000015. doi: 10.1371/journal.pdig.0000015 (PMC9931335; doi:10.1371/journal.pdig.0000015)
Supplement: Attachment — Submitted filename: Response letter.docx [file pdig.0000015.s004.docx]

29^th^ October 2021

Valentina Lichtner
Academic Editor
PLOS Digital Health

Dear Dr Lichtner,

Thank you for considering our manuscript entitled “Digital health use in gestational diabetes mellitus on maternal and neonatal outcomes: A systematic review and meta-analysis of randomised controlled trials” to PLOS Digital Health. As proposed by the reviewers and editors, we have made substantial changes to our manuscript. We hope that these will meet the requirements of the journal.

**The editor had the following 5 suggestions.**

**Suggestion #1. restructuring the paper, placing the methods section after the introduction/before the findings;**

Thank you for the suggestion. We have restructured our manuscript to place the methods section after the introduction, just before the findings as proposed.

**Suggestion #2 providing further details on previous systematic reviews, as recommended by both reviewers.**

We note on the suggestion. We have included additional details of the previous systematic reviews in the introduction as follows:-

*Several reviews have suggested that there were limited benefits of using digital health in women with GDM. Several reviews have suggested that there were limited benefits of using digital health in women with GDM. In one of the earliest systematic review on the use of telemedicine for people with GDM in 2015, Rasekaba et al in their meta-analysis of 3 studies reported that telemedicine had no beneficial impact on maternal outcomes. However, an updated review by Ming and colleagues of seven randomized controlled studies reported that telemedicine was useful to reduce HbA1c but not maternal and neonatal outcomes. Since the publication of these reviews, several new trials have been conducted which have reported otherwise.*

We have also included additional details to discuss on previous work in our discussion section as follow:-

**Comparison with existing literature**

*Studies and reviews performed to date have shown that digital health can be used to facilitate clinical support, monitoring of care as well as capacity building. O’Brien in one of the earliest review on this topic examined how technology can be used to support lifestyle interventions in pregnant women. They found that technology had the potential to support current lifestyle interventions in pregnancy. Nevertheless, similar to our study, they noted that the evidence base were weak, due to the lack of studies as well as poor evidence base. A more focused review by Ming and colleagues comparing digital health technology in GDM found that digital health resulted in modest improvement in maternal glycaemic control. Similar to the study by O’Brien, the authors noted only few randomized controlled studies that have been conducted to date. A more updated review by Garg et al in 2020 examined the impact of mHealth use on people with GDM. In their review of 11 studies, the authors reported that mHealth could be useful to enhance patient care and support self-management. Similar to these studies, our review on a broader topic encompassing different technologies found that digital health can be effective in improving maternal outcomes*

**Suggestion #3. providing additional analysis or comments on participants' adherence. Both reviewers’ comment on this, e.g. Reviewer 2 asks about studies that did not report frequency/engagement in glucose monitoring.**

We take note of the suggestion by the editor. We have expanded this in our results to state the following

*Eight studies also reported participants adherence to study protocol. However, the definitions used varied depending on studies. This includes the total number of glucose monitoring performed as recommended in the study, glucose monitoring frequency, or compliance to trial recommendations (e.g. dietary advice, physical activities or glucose monitoring). Due to the heterogeneity of definitions, pooling was not performed.*

Unfortunately, due to the heterogeneity of definitions used, we were unable to perform any subgroup analysis or stratify the studies based on the compliance. We acknowledge that this will be an important parameter to assess which may explain the results. To clarify this, we have also included a brief description of how this important parameter can affect the outcomes in our discussion as follow.

*Poor compliance has been associated with poor glycaemic control. Given the central importance for people with GDM to self-manage, it was surprising that very few studies adequately quantified compliance of their study participants to the recommendations, with an overall compliance rate of 66% to 85%, depending on definitions used. This may stem from the lack of a standardised definition to report study compliance, unlike studies reporting on medication adherence where adherence is often defined when a person is compliant to at least 80% of prescribed therapy. Given the importance of this, we recommend that future trials ensure that these data are captured and reported.*

**Suggestion #4. Explaining the different digital health interventions included in the studies, e.g. similarities and differences between mhealth, apps, social platforms,.., also in light of both reviewers’ comments.**

 We thank the editor for pointing this very important observation. We have discussed this point in our revised discussion as follow

*The management of GDM is a very time-consuming activity, for both healthcare providers and patients. Digital health can help in educating women, improve self-care through nutritional and exercise advice, improve monitoring adherence and possibly improve the care of women with GDM. As such, in our analysis, we combined the various types of digital health interventions used by researchers in our meta-analysis, including telehealth (providing clinical services from a distance), mHealth (use of mobile phones for remote connection and transmission of clinical data to provider for feedback), and patient platforms (using interactive platform to support and facilitate clinical care). We took this approach as we believe that the ultimate goal are similar, which is to improve GDM care. This wide variation of practice may have contributed to some of the observed heterogeneity, and can be partly explained from the subgroup analyses.*

*The only digital health that was significantly associated with additional improved glycaemic control was the use of social platform and mHealth. This may be due to the nature of the intervention, which often involves the provision of education, monitoring and motivation by peers as well as healthcare providers. The combination of these activities could have resulted in an increase the level of interaction and thus compliance to advice by the healthcare providers. However, for other outcomes such as maternal and neonatal outcomes, these were not apparent with digital health use. We offer several possible reasons for the lack of additional benefits of using digital health on GDM. Firstly, most of the outcomes reported such as caesarean rates, admission to intensive care unit are influenced directly by local clinical practice, and may not be directly influenced by intervention. Secondly, these interventions are inherently prone to selection bias, as women with easy accessibility to electronic devices are able to participate in the trials. As these information were not reported in all studies, it would be difficult assess how applicable these results would be to the wider general population, and thus the suggested benefits are speculative.*

*Other digital health that have not been thoroughly examined is the use of social platforms and game based support in GDM. Social media sites such as Facebook or Twitter use has increased dramatically over the past few years especially among the younger population. While there is some evidence on the effects of social media on health behaviour outcomes, our review did not find any trials that were conducted to date. In addition, the use of gamification has been shown to be useful to target behavioural outcomes, especially to increase physical activity and encourage medication adherence. In this review, we only found one study which attempted to use Nintendo Wii to encourage physical activities among women with GDM (NCT03073551). As such, additional scientific inquiry and evidence is needed before any digital health can be recommended as a viable means to improve self-care in GDM.*

**Suggestion #5. Please also copy-edit the manuscript, checking grammar and typos.**

We take note and apologise for the oversight. We have edited our manuscript and spell-checked to ensure there are no typographical errors.

**Reviewer #1 had the following comments.**

**Comment #1: I thank the author for this timely investigation into the use of technologies for the management of GDM among pregnant women. I found the article well written, with results well presented, though with the occasional typo and the methods section in the wrong location.**

We thank the reviewer for the encouraging comments. We have spell-checked the manuscript to ensure that there are no more typographical errors. As suggested, we have also moved the methods section and placed it after the introduction, before the findings section. We also updated our search to October 31^st^, 2021 to ensure the currency of our review.

**Comment #2. The review claims to have been performed to update existing findings from three cited reviews (referred to as "several" reviews). However, we are not presented with the results of these reviews, nor the studies included. Just that there was "limited support". At a minimum it would be good to know the year of publication and types of interventions included along with number of studies. At best we could read actual statistical results. This could give some indication of the number or types of studies that were reviewed here and not before.**

We note on the suggestion. We have included additional details of the previous systematic reviews as follow in the introduction:-

*Several reviews have suggested that there were limited benefits of using digital health in women with GDM. Several reviews have suggested that there were limited benefits of using digital health in women with GDM. In one of the earliest systematic review on the use of telemedicine for people with GDM in 2015, Rasekaba et al in their meta-analysis of 3 studies reported that telemedicine had no beneficial impact on maternal outcomes. However, an updated review by Ming and colleagues of seven randomized controlled studies reported that telemedicine was useful to reduce HbA1c but not maternal and neonatal outcomes. Since the publication of these reviews, several new trials have been conducted which have reported otherwise.*

**Comment #3. Adherence to the interventions is an absolute key variable when talking about non-face-to-face interventions. This factor is only briefly mentioned with a single line informing the reader that adherence was at 66-85% across studies. I would have thought this to warrant greater attention, and potentially a sensitivity analysis. At a minimum this is a topic to be included in the discussion.**

We thank the reviewer for the suggestion. We have expanded this in our results to state the following

*Eight studies also reported participants adherence to study protocol. However, the definitions used varied depending on studies. This includes the total number of glucose monitoring performed as recommended in the study, glucose monitoring frequency, or compliance to trial recommendations (e.g. dietary advice, physical activities or glucose monitoring). Due to the heterogeneity of definitions, pooling was not performed.*

Unfortunately, due to the heterogeneity of definitions used, we were unable to perform any subgroup analysis or stratify the studies based on the compliance. We acknowledge that this will be an important parameter to assess which may explain the results. To clarify this, we have also included a brief description of how this important parameter can affect the outcomes in our discussion as follow.

*Poor compliance has been associated with poor glycaemic control. Given the central importance for people with GDM to self-manage, it was surprising that very few studies adequately quantified compliance of their study participants to the recommendations, with an overall compliance rate of 66% to 85%, depending on definitions used. This may stem from the lack of a standardised definition to report study compliance, unlike studies reporting on medication adherence where adherence is often defined when a person is compliant to at least 80% of prescribed therapy. Given the importance of this, we recommend that future trials ensure that these data are captured and reported.*

**Comment#4. The study quality was assessed and is clearly an issue. Would the authors consider highlighting the few very good quality studies and their findings? Or a sensitivity analysis by quality for the results?**

We thank the reviewer for the suggestion. We have conducted a subgroup analysis as proposed by the reviewers and only considered those which were high-quality. We found that results were changed and that digital health now had minimal impact on both maternal and neonatal outcomes. We have now included this in our results as follows:-

*We also examined if results differed when high-quality studies were only included in our analyses. Results showed that digital health had minimal impact on all maternal and neonatal outcomes after inclusion of only high-quality studies*

We have also included a short discussion on this results as follows:-

*However, before digital health is widely implemented in routine practice, clinicians will also need to take into consideration the quality of evidence found in this review, which was rated to be mostly moderate to very low, as well as the limitations of trials which were mostly very small in sample sizes, with varying levels of care. In our subgroup analyses of only high-quality trials, these results were not significantly different from usual care.*

**Comment #5. The included interventions are referred to as "digital health" but are clearly very varied. There should be some discussion of their current relevance. Clearly there is a shift toward the use of apps but are earlier interventions still used, any fading away?**

We take note of this comment. We have made some brief discussion on this in our revised discussion as follow

*Social media sites such as Facebook or Twitter use has increased dramatically over the past few years especially among the younger population. While there is some evidence on the effects of social media on health behavior outcomes, our review found only limited trials that were conducted to date. Most of the studies have mainly focused on the use of health applications, perhaps due to the popularity of such applications and high smartphone ownerships worldwide.*

**Reviewer #2 had the following comments**

**Comment #1. This is important and timely research given the increasing public health importance of GDM and the rise of digital technologies in maternal health**.

We thank the reviewer for the encouraging comments.

**Comment #2. This statement late in the article very nicely sums up the contribution of this article: "This review suggest that the use of digital health can be a useful adjunct to supplement the care of pregnant women with GDM, as they result in similar maternal and neonatal outcomes without any adverse outcomes."**

We thank the reviewer for the encouraging comments.

**Comment #3.  To the point about timeliness, 9 of the 26 included studies were published after 2019. Further 16/26 were published since 2018, and 23/26 were published since 2015. This speaks to the need for this type of a systematic review and meta-analysis now. The authors may consider including a statement - or even a figure - that shows how much of this systematic review is focused on just the last few years.**

We thank the reviewer for the suggestion. We have included a statement in our discussion to highlight this very important observation as suggested. The revised results reads as follow:-

*The included studies were published between 2007-2021, with more than half (n=18; 64.3%) of studies published within the past five years*

We have also included a short discussion of these important observation as follow:-

*Our study found that over the past few years, there has been an explosion of studies that have examined the use of digital health for GDM, with eighteen studies published to date. This is not surprising, given the rapid evolution of electronics, where most families would have at least one device that can be used to provide communication between patients and providers. Indeed, recent studies have reported on how digital health are used to support people with various chronic conditions such as hypertension and diabetes.*

**Comment #4. There were a few places in the article where more context would be helpful: A citation indicating that GDM is the most common medical complication of pregnancy**

We thank the reviewer for the pointing this very important observation. We have now included the additional citation for the statement as suggested by the reviewer. Our revised sentence now reads as follow:-

*Gestational diabetes mellitus (GDM) is an increasingly common diagnosis during pregnancy and has a substantial effect on maternal and foetal morbidity including risk of developing preeclampsia, shoulder dystocia, caesarean delivery as well as future health complications such as cardiovascular diseases, type 2 diabetes and cancer risk.*

**Comment #5. Is there any data the authors can include that help the reader understand the global year-over-year increase in GDM? This is alluded to on line 159 on p24.**

We thank the reviewer for the suggestion. Unfortunately, there are no global data to provide readers on the prevalence of GDM on a year-over-year due to the heterogeneity of definitions used as well as other patient related factors such as ethnicity and lifestyle. However, we have edited our introduction to include additional details as follow.

The prevalence of GDM was estimated to be 8-9% of all pregnancies, and is rising due to an increased rate of obesity, changing threshold of GDM as well as lifestyle changes such as physical inactivity and the adoption of modern lifestyles. Women with GDM have a significant increase lifetime risk of developing type 2 diabetes, and a three-fold increase in developing metabolic syndrome and cardio-vascular diseases.

**Comment #6  Were there any policies around diabetes that could have been discussed to give a better scope of the problem?**

We thank the reviewer for the suggestion. We have now included a short discussion around the policies on treating diabetes to provide readers a better context and scope of the issue.

*Current guidelines have recommended the need for post-partum follow up and care, including continued support for lifestyle changes. In women with GDM, medical nutrition therapy remains the mainstay treatment with daily self-monitoring of blood glucose (SMBG), aimed at normalizing blood glucose to reduce the risk of complications as well as improve maternal and foetal outcomes as well as risk for developing metabolic syndromes in the future.*

**Comment #7. The authors could have gone into a bit more detail about why they specifically think that this work can reduce the need for women to advocate for themselves to specialist care.**

We thank the reviewer for the suggestion. We have now included a short discussion to clarify why we feel digital health can be useful for women with GDM in our revised introduction. Our introduction now reads as follows:-

*Technological innovations have provided opportunities for novel approaches to improve the care of people with diabetes and women with GDM. Some potential benefits of using technology include the ability to provide support and immediate feedback, reducing the distance barriers as well as reduce healthcare costs through resource pooling. Multiple reviews and trials performed to date have suggested that digital health, defined as electronic systems designed to provide services remotely to support clinical decisions can be a useful adjunct to supplement and enhance patient care, especially among those with chronic conditions. The premise is that users of digital health have an increased interaction with health professional, and helps patients become more engaged in managing their own health condition. Some examples of digital health used in diabetes care include telehealth, game-based support, mobile health (mHealth) as well as patient portals. Digital health can be an important solution especially for women with GDM, given the limited time clinicians have to manage and educate these women who requires short-term adjustment to their therapy. More importantly, digital health can help ensure that health resources are optimally utilised.*

**Comment #8. Various technologies and use cases were pooled together for this systematic review and meta-analysis. Ten studies of the 26 indicate NR for monitoring frequency. It was unclear to me if this means they did not report frequency or that they were not engaged in regular glucose monitoring.**

We agree this was unclear in our initial submission The monitoring frequency was frequency as proposed by each individual study. Some studies only described those participants were required to monitor their blood glucose levels but did not specify the frequency, while other had explicitly described the frequency each week. To clarify this point, we have now edited to change the heading to state it is the monitoring frequency in trial instead. We have also included this in our results to clarify this.

*The frequency of monitoring varied between studies, and could range from once daily up to six times a day (Table 1).*

**Comment #9. Was there a distinction between monitoring and purely educational interventions? Many in the research community talk about digital health as a single intervention, but there is more nuance to it. We are increasingly understanding this. This is not a suggestion to change the analysis, but rather to provide some clarity on the reasoning for including such a diverse set of digital health interventions.**

We agree and thank the reviewer for pointing this out. As the reviewer had pointed out, there is various definitions of digital health, and that it could involve only monitoring or educational, or a combination of these. This is described in our discussion section as follow:-

*The management of GDM is a very time-consuming activity, for both healthcare providers and patients. Digital health can help in educating women, improve self-care through nutritional and exercise advice, improve monitoring adherence and possibly improve the care of women with GDM. As such, in our analysis, we combined the various types of digital health interventions used by researchers in our meta-analysis, including telehealth (providing clinical services from a distance), mHealth (use of mobile phones for remote connection and transmission of clinical data to provider for feedback), and patient platforms (using interactive platform to support and facilitate clinical care). We took this approach as we believe that the ultimate goal are similar, which is to improve GDM care. This wide variation of practice may have contributed to some of the observed heterogeneity and can be partly explained from the subgroup analyses.*

We have included this into our study limitation as follow

*There was considerable variation in the types of digital health technology used, the level of care participants received as well as study duration. These variation GDM varies depending on study regions and countries could have contributed to the observed heterogeneity and explain the difference in results reported by these studies. In addition, we had also pooled studies using different technologies such as smartphone apps, telemonitoring devices together in our analyses. While this may not be ideal, the objective of these studies was to improve glycaemic control and support patient self-efficacy. As such, we took a broader view of this and pooled the results together, and stratified this in our analyses*

**Comment #10. "The only digital health that was significantly associated with additional improved glycaemic control was the use of social platform and mHealth. " This is a key finding, perhaps *the* key finding. But it was not discussed. Please discuss why this might be and perhaps look to the reviewed articles for insight to share with the reader. This will have implications for both further research and practice.**

We thank the reviewer for pointing this very important observation. We have now included this into our discussion as follows:-

*The only digital health that was significantly associated with additional improved glycaemic control was the use of social platform and mHealth. This may be due to the nature of the intervention, which often involves the provision of education, monitoring and motivation by peers as well as healthcare providers. The combination of these activities could have resulted in an increase the level of interaction and thus compliance to advice by the healthcare providers. However, for other outcomes such as maternal and neonatal outcomes, these were not apparent with digital health use. We offer several possible reasons for the lack of additional benefits of using digital health on GDM. Firstly, most of the outcomes reported such as caesarean rates, admission to intensive care unit are influenced directly by local clinical practice, and may not be directly influenced by intervention. Secondly, these interventions are inherently prone to selection bias, as women with easy accessibility to electronic devices are able to participate in the trials. As this information were not reported in all studies, it would be difficult assess how applicable these results would be to the wider general population, and thus the suggested benefits are speculative.*

**Comment #11. I would like to see the authors speak to the other systematic reviews in this space: Ming et al. JMIR 2016; Garg et al. JDST 2020; O'Brien et al. EJCN 2014. The authors mention Ming, but do not discuss that article in a substantive way. The other two articles are not mentioned. (I am not an author on any of these articles, nor do I have any affiliations with them.) It is important to give the reader the full context because they may not know this literature. It's also important to acknowledge the work of others, even if there are limitations in the work. See my earlier comment about the timeliness of this review, and the recency of included studies.**

Thank you for the suggestion to improve our manuscript. We have now included additional information on the work of other authors that have been performed to date. The revised paragraph now reads as follow

*Several reviews have suggested that there were limited benefits of using digital health in women with GDM. In one of the earliest systematic review on the use of telemedicine for people with GDM in 2015, Rasekaba et al in their meta-analysis of three randomised controlled studies reported that telemedicine had no beneficial impact on maternal outcomes. However, an updated review by Ming and colleagues of seven studies reported that telemedicine was useful to reduce HbA1c but not maternal and neonatal outcomes. Nevertheless, these reviews are only specific to a single telemedicine technology and do not address the various digital health interventions that have been used to date. In addition, since the publication of these reviews, several new trials have been conducted, which warrants an update of the results of these reviews.*

We have also included additional details to discuss on previous work in our discussion section as follow:-

**Comparison with existing literature**

*Studies and reviews performed to date have shown that digital health can be used to facilitate clinical support, monitoring of care as well as capacity building. O’Brien in one of the earliest review on this topic examined how technology can be used to support lifestyle interventions in pregnant women. They found that technology had the potential to support current lifestyle interventions in pregnancy. Nevertheless, similar to our study, they noted that the evidence base were weak, due to the lack of studies as well as poor evidence base. A more focused review by Ming and colleagues comparing digital health technology in GDM found that digital health resulted in modest improvement in maternal glycaemic control. Similar to the study by O’Brien, the authors noted only few randomized controlled studies that have been conducted to date. A more updated review by Garg et al in 2020 examined the impact of mHealth use on people with GDM. In their review of 11 studies, the authors reported that mHealth could be useful to enhance patient care and support self-management. Similar to these studies, our review on a broader topic encompassing different technologies found that digital health can be effective in improving maternal outcomes*

**Comment #12. There were a dozen or so grammatical issues throughout. That is fully to be expected on a manuscript of this length. We have captured some of them in this section. We suggest a couple copy-editing read-throughs before publication.**

We take note and have copy-edited our manuscript.

**Comment #13. throughout: be consistent with m-health, mHealth. define on first use.**

We take note and have ensured that the term mHealth is used throughout the whole manuscript. We have also defined this term on first use. Detailed explanation of each of the digital health terms can also be found in the Appendix for the benefits of the readers.

**Comment #14. cover page: “A high certainty of evidence…“ This statement in the abstract was unclear. This is also on lines 45-47 on p5.**

We have revised this statement to read as follow

*Among those randomized to digital health, there was a lower need for caesarean delivery (Relative risk: 0.81; 0.69 to 0.95; high certainty) and foetal macrosomia (0.67; 0.48 to 0.95; high certainty).*

**Comment #15. p4: lines 78-79 be more explicit about why we need to reduce the need for women to utilize step-up care.**

We agree this was unclear. We have deleted the statement instead due to the low quality of evidence available to avoid any confusion. Our revised sentence now read as follows:-

*This review provides support for using digital health to support women with gestational diabetes in their care.*

**Comment #16. p18: line 13 change "participant" to "participants"**

We thank the reviewer for pointing this out.

*Six studies reported that participants were satisfied with using digital health to monitor their blood glucose, due to the ability to facilitate communication with healthcare providers*

**Comment #17. "we found that tele-education had a larger impact on glycaemic control, but this was mainly due to the small number of studies examining this form of intervention." -- this statement requires further explanation**

We thank the reviewer for pointing this out. We have expanded our explanation of this in our revised results as follows:-

*When we explored whether the type of activity delivered (either telemonitoring or tele-education) had any impact on maternal and neonatal outcomes, we found that tele-education had a larger impact on glycaemic control. This could be partly explained by the small number of studies examining this form of intervention which had reported a large effect size.*

**Comment#18. line 170 explain what “inappropriate self care behavior” looks like**

We agree this was unclear. We have deleted the statement instead to avoid any confusion.. Our revised sentence now read as follows:-

*Indeed, in many of these trials, the human-computer interaction is often overlooked, due to poor study design which lacked a theoretical framework.*

**The editorial team have made the following comment.**

**Comment #1. We have noticed that you have cited 4 in the manuscript file. However, there are no corresponding files uploaded to the submission. Please ensure that all files are present to ensure that your paper is fully reviewed.**

We have included all relevant files in our revised manuscript. We apologise for the oversight.

We thank the reviewers and the editor for their invaluable time to review our manuscript, which has significantly imporved our manuscript quality. We trust that these changes will be acceptable to the reviewers and editor.

Yours truly

Shaun LEE
on behalf of the authors
