# Supplementary material for: Digital health interventions for gestational diabetes mellitus: A systematic review and meta-analysis of randomised controlled trials
Source: PLOS Digit Health. 2022 Feb 24;1(2):e0000015. doi: 10.1371/journal.pdig.0000015 (PMC9931335; doi:10.1371/journal.pdig.0000015)
Supplement: Attachment — Submitted filename: Response to reviewer.docx [file pdig.0000015.s005.docx]

29 November 2021

Liliana Laranjo
Section Editor
PLOS Digital Health

Dear Dr Liliana Laranjo

We are pleased to resubmit our revised manuscript entitled " Digital health interventions for gestational diabetes mellitus: A systematic review and meta-analysis of randomised controlled trials " to the PLOS Digital Health As suggested by the editor, we have revised our manuscript based upon the suggestions.

The editor had the following comments.

**Comment #1. Please review your reference list to ensure that it is complete and correct. If you have cited papers that have been retracted, please include the rationale for doing so in the manuscript text, or remove these references and replace them with relevant current references. Any changes to the reference list should be mentioned in the rebuttal letter that accompanies your revised manuscript. If you need to cite a retracted article, indicate the article’s retracted status in the References list and also include a citation and full reference for the retraction notice.**

We have reviewed our reference list to ensure it is complete.

**Comment #2**. **The definition of digital health provided in the introduction seems to focus on clinicians (e.g., “support clinical decisions”) and telemonitoring/telehealth (e.g., “The premise is that users of digital health have an increased interaction with health professionals”). Digital health is much broader than this and does not have to involve the clinician, it can solely support patients/consumers too. Suggest using a different definition and providing an appropriate reference. The definition provided in the methods is much better than the one in the introduction.**

We take note of the suggestion. We have edited our introduction to include a broader definition as suggested and used the definitions by the World Health Organisation. Our revised paragraph in the Introduction, Line 104 now reads as follows:-

*Existing literature reviews performed to date on digital health interventions, defined as electronic systems in medicine and other health professions designed to provide services remotely to manage illness and health risk and promote wellness is key towards universal health coverage, as it provides safe, timely and affordable access to health services for all. The premise is that digital health interventions can facilitate training, surveillance, and service delivery, and more importantly empower users of digital health to make better informed decisions about their own health in new and innovative ways. Some examples of digital health interventions used in diabetes care include telehealth, game-based support, mobile health (mHealth) as well as patient portals. Digital health interventions can be an important solution especially for women with GDM, given the limited time clinicians have to manage and educate these women who requires short-term adjustment to their therapy. More importantly, digital health interventions can help ensure that health resources are optimally utilised.*

**Comment #3** Th**is sentence is missing a reference: “More importantly, digital health can help ensure that health resources are optimally utilised.”**

We apologise for the oversight. We have now included a reference to support that statement.

- Lee JY, Lee SWH. Telemedicine Cost–Effectiveness for Diabetes Management: A Systematic Review. Diabetes Technology & Therapeutics. 2018;20(7):492-500

**Comment #4. Sentence is repeated: “Several reviews have suggested that there were limited benefits of using digital health in women with GDM”.**

We apologise for the oversight. We have removed the repeating sentence as suggested.

**Comment #** 5. **“Several reviews have suggested that there were limited benefits of using digital health in women with GDM”.: These reviews are specifically focused on telemedicine so the sentence should say telemedicine instead of digital health.**

We take note and thank the editor for the suggestion. We have now edited the sentence to state the following.
*Several reviews have suggested that there were limited benefits of telemedicine in women with GDM*

**Comment #6. The introduction should also mention the Garg et al 2020 systematic review.**

We thank the reviewer for the suggestion. We have included the review in our revised introduction as suggested.

*Several reviews have suggested that there were limited benefits of telemedicine in women with GDM. In one of the earliest systematic review on the use of telemedicine for people with GDM in 2015, Rasekaba et al in their meta-analysis of 3 studies reported that telemedicine had no beneficial impact on maternal outcomes. However, an updated review by Ming and colleagues of seven randomised controlled studies reported that telemedicine was useful to reduce HbA1c but not maternal and neonatal outcomes. Garg et al in their recent review in 2020 attempted to clarify this association, and reported that mobile apps along with medical care could be effective to manage and prevent risk associated with GDM.*

**Comment #7.** **There is a problem with the references in this excerpt: “Several reviews have suggested that there were limited benefits of using digital health in women with GDM.[13-15] In one of the earliest systematic review on the use of telemedicine for people with GDM in 2015, Rasekaba et al in their meta-analysis of 3 studies reported that telemedicine had no beneficial impact on maternal outcomes.[14]However, an updated review by Ming and colleagues of seven randomised controlled studies reported that telemedicine was useful to reduce HbA1c but not maternal and neonatal outcomes.[13]”**

We apologise for the error. We have updated the references for the excepts as follows:-

*Several reviews have suggested that there were limited benefits of telemedicine in women with GDM. In one of the earliest systematic review on the use of telemedicine for people with GDM in 2015, Rasekaba et al in their meta-analysis of 3 studies reported that telemedicine had no beneficial impact on maternal outcomes. However, an updated review by Ming and colleagues of seven randomised controlled studies reported that telemedicine was useful to reduce HbA1c but not maternal and neonatal outcomes.*

**Comment #8. Can you rephrase and better clarify the aim: “we conducted a systematic review and meta-analysis to determine the innovative use of technology in supporting routine care delivery in women with GDM.”**

We agree this was unclear. We have revised the sentence in Line 135 to clarify this point. The revised sentence now reads:-

*In light of these new developments and to address these important knowledge gaps, we conducted a systematic review and meta-analysis to investigate the efficacy of digital health interventions use to support women with GDM.*  

**Comment #9. Please provide references to the definitions provided in Box for different digital health types. Given that several analyses focus on this variable, these definitions should be provided in the main manuscript.**

We thank the editor for the suggestion. We have now moved the definitions to the main text as suggested and included the references for these definitions.

**Comment #10. Please clarify in the methods that interventions could be classified as involving more than one digital health type.**

We agree this was unclear in our initial submission. Interventions in the current review were only classified as one digital health type in the current study. This is detailed in our revised methods as follows:-

*As the technologies used varied within each study, we followed the approach taken by previous reviews and classified these as one of the following:- telehealth, mobile health or mHealth (where mobile devices are used to support medical care), game-based support, social platforms and patients portal (see Table 1 for full definitions).*

We acknowledge that this is a potential limitation of this review. As such, we have also included this as a study limitation in our discussion

*There was considerable variation in the types of digital health technology used, the level of care participants received as well as study duration. Similarly, there was variation in the type of intervention used by each trial. For example, some trials could have included elements of education and monitoring. However, to simplify our classification and reduce any chance of misclassification, we classified the intervention based upon the primary aim of the study, depending on whether it was to provide education or to improve blood glucose monitoring. These variations in classification and GDM definition depending on study regions and countries could have contributed to the observed heterogeneity and explain the difference in results reported by these studies.*

**Comment #11. Please clarify whether an intervention could deliver both telemonitoring and teleeducation. Did you analyse multicomponent interventions?**

We agree this was unclear in our initial submission. We had classified the intervention based upon the primary aim of each trial. While we did consider classifying a trial as a multicomponent intervention, the aim of the study was determine the efficacy of different digital health interventions. To clarify this, we have included this into the methods as follows;-

*Intervention Classification:*

*Intervention and control conditions were subsequently classified into either one of the following categories based upon an adaptation of the definitions from the American Telemedicine Association (2016) reported previously by Lee et al.*

*●	Tele-education: Any intervention aimed at educating teaching, or training patients remotely using live interactive streaming or by stored educational material.*

*●	Telemonitoring: Any process which allows for the delivery and/or exchange of information to monitor a health status of patients remotely.*

*The classification of intervention was based upon the primary aim of each trial, such as to address the lack of monitoring or to provide health related education. For example, if the digital health intervention targets to educate a patient on the importance of blood glucose monitoring but does not allow for exchange of information or feedback, this was classified as tele-education. In comparison, if the trial request that participants monitor their blood glucose levels and provides feedback based upon these readings, the intervention was classified as a telemonitoring study. Trials that included both components of education and monitoring delivered remotely to empower their patients, these trials were classified as tele-education and telemonitoring studies.*

We acknowledge that this is a potential limitation of this review. As such, we have also included this as a study limitation in our discussion

*There was considerable variation in the types of digital health technology used, the level of care participants received as well as study duration. Similarly, there was variation in the type of intervention used by each trial. For example, some trials could have included elements of education and monitoring. However, to simplify our classification and reduce any chance of misclassification, we classified the intervention based upon the primary aim of the study, depending on whether it was to provide education or to improve blood glucose monitoring. These variations in classification and GDM definition depending on study regions and countries could have contributed to the observed heterogeneity and explain the difference in results reported by these studies. In addition, we had also pooled studies using different technologies such as smartphone apps, telemonitoring devices together in our analyses. While this may not be ideal, the objective of these studies was to improve glycaemic control and support patient self-efficacy. As such, we took a broader view of this and pooled the results together and stratified this in our analyses*

**Comment #12. This sentence needs to be rephrased, it is unclear what you mean by “only social platform”, and what you are comparing social platforms with: “Subgroup analysis showed that only social platform was associated with better glycaemic control among mothers, lower rates of caesarean delivery and weight gain throughout pregnancy.”**

We agree this was unclear. We have now rephrased this to include also quantitative results and referred the readers to the corresponding table as suggested. Our revised paragraph now reads as follows:-

*Subgroup analysis showed that the use of social platform was associated with better fasting plasma glucose control among mothers (-1.09 mmol/L; 95% CI: -1.40 to -0.78; , I^2^: 77%, 4 studies), postprandial glucose (-1.35 mmol/L; -2.05 to -0.65, I^2^: 90%, 4 studies) and HbA1c (-1.17%, -1.67 to -0.67, 1 study) compared to routine care at the end of study. In addition, there was lower rates of caesarean delivery (Risk ratio: 0.52, 0.38 to 0.72, I^2^: 0%, 4 studies) and weight gain (-4.71kg; -5.46 to -3.96, 1 study) throughout pregnancy compared to routine care (Table 3). Social platform use was also found to reduce the rates of foetal macrosomia (0.22; 0.08 to 0.60, I^2^: 0%, 3 studies) and hypoglycaemia among new-borns (0.20; 0.05 to 0.77, I^2^: 0%, 3 studies, Table 4). Both mHealth (0.79, 0.67 to 0.92, I^2^: 38%, 10 studies) and social platforms (0.52; 0.38 to 0.72, I^2^: 0%, 4 studies) were effective in reducing the rates of caesarean delivery. There were fewer neonates who required the use of the neonatal intensive care unit among women randomised to mHealth compared to routine care (0.76; 0.46 to 1.28, I^2^: 42%, 6 studies, Table 4).*

**Comment #13. For the subgroup analysis on the social platforms please provide quantitative results and refer the readers to corresponding tables/supplements.**

We agree this was unclear. We have now rephrased this to include also quantitative results and referred the readers to the corresponding table as suggested. Our revised paragraph now reads as follows:-

*Subgroup analysis showed that the use of social platform was associated with better fasting plasma glucose control among mothers (-1.09 mmol/L; 95% CI: -1.40 to -0.78; , I^2^: 77%, 4 studies), postprandial glucose (-1.35 mmol/L; -2.05 to -0.65, I^2^: 90%, 4 studies) and HbA1c (-1.17%, -1.67 to -0.67, 1 study) compared to routine care at the end of study. In addition, there was lower rates of caesarean delivery (Risk ratio: 0.52, 0.38 to 0.72, I^2^: 0%, 4 studies) and weight gain (-4.71kg; -5.46 to -3.96, 1 study) throughout pregnancy compared to routine care (Table 3). Social platform use was also found to reduce the rates of foetal macrosomia (0.22; 0.08 to 0.60, I^2^: 0%, 3 studies) and hypoglycaemia among new-borns (0.20; 0.05 to 0.77, I^2^: 0%, 3 studies, Table 4). Both mHealth (0.79, 0.67 to 0.92, I^2^: 38%, 10 studies) and social platforms (0.52; 0.38 to 0.72, I^2^: 0%, 4 studies) were effective in reducing the rates of caesarean delivery. There were fewer neonates who required the use of the neonatal intensive care unit among women randomised to mHealth compared to routine care (0.76; 0.46 to 1.28, I^2^: 42%, 6 studies, Table 4).*

**Comment #14. Please provide quantitative results: “Results showed that digital health had minimal impact on all maternal and neonatal outcomes after inclusion of only high-quality studies.”**
We apologise for the oversight and confusion. We have now created a supplementary table and referred this in our revised results. The revised sentence now reads as follows:-

*We also examined if results differed when high-quality studies were only included in our analyses. Results showed that the use of digital health interventions had minimal impact on all maternal and neonatal outcomes after inclusion of only high-quality studies (S3 Table).*

**Comment #15. The main findings section of the Discussion should only report findings from your study. The following needs to be deleted: “Digital health are thought to have a profound effect to enhance health services delivery, enabling new models of care and shifting the focus of the health systems towards person-centre care.[12, 63]”**

We take note of the suggestion. We have removed the statement as suggested and ensured that we only report findings from our study.

**Comment #16. The following is an incorrect interpretation. Just because there were no significant differences does not mean that they are similar. “In terms of adverse neonatal outcomes such as risk of hypoglycaemia and need for neonatal intensive care unit hospitalization, results were similar to routine care, suggesting that supplementing or replacing clinic face-to-face visit with digital health was possible.”**
We agree and thank the editor for pointing this out. We have now edited the paragraph to clarify this. The revised sentence now reads as follows:-

*In this review, we noted that the use of digital health could improve maternal glycaemic outcomes such as fasting plasma glucose and HbA_1c_ as well as delivery outcomes including foetal macrosomia and need for caesarean delivery. In terms of adverse neonatal outcomes such as risk of hypoglycaemia and need for neonatal intensive care unit hospitalization results were similar to routine care. Taken together, this systematic review and meta-analysis provides some evidence to suggest that digital health could be a useful adjunct to routine care.*

**Comment #17**. Rephrase to remove only, as there were two types of digital health interventions that were significantly associated with additional improved glycaemic control : “The only digital health that was significantly associated with additional improved glycaemic control was the use of social platform and mHealth.”

We agree and thank the editor for pointing this out. We have now edited the paragraph to clarify this. The revised sentence now reads as follows:-

*The meta-analysis results suggest that intervention which involves the provision of education, monitoring and motivation by peers as well as healthcare providers were associated with improved glycaemic control. The combination of monitoring and education could have resulted in an increase the level of interaction between participants and healthcare providers and thus compliance to treatment advice.*

**Comment #18**. **Can you clarify what you mean and rephrase: “The combination of these activities could have resulted in an increase the level of interaction and thus compliance to advice by the healthcare providers.”**
We agree and thank the editor for pointing this out. We have now edited the paragraph to clarify this. The revised sentence now reads as follows:-

*The meta-analysis results suggest that intervention which involves the provision of education, monitoring and motivation by peers as well as healthcare providers were associated with improved glycaemic control. The combination of monitoring and education could have resulted in an increase the level of interaction between participants and healthcare providers and thus compliance to treatment advice.*

**Comment #19.**. **The following reasons would make sense if these were non-randomized studies, which is not the case: “However, for other outcomes such as maternal and neonatal outcomes, these were not apparent with digital health use. We offer several possible reasons for the lack of additional benefits of using digital health on GDM. Firstly, most of the outcomes reported such as caesarean rates, admission to intensive care unit are influenced directly by local clinical practice, and may not be directly influenced by intervention. Secondly, these interventions are inherently prone to selection bias, as women with easy accessibility to electronic devices are able to participate in the trials. As these information were not reported in all studies, it would be difficult assess how applicable these results would be to the wider general population, and thus the suggested benefits are speculative.”**

We agree this was unclear. We have removed this from our revised manuscript to avoid any confusion as suggested.

**Comment #20 Provide references: “While there is some evidence on the effects of social media on health behaviour outcomes”**

We take note of the editor’s suggestion. We have included the references to support the statement as suggested. These references are listed as follow:-

- Petkovic J, Duench S, Trawin J, Dewidar O, Pardo Pardo J, Simeon R, et al. Behavioural interventions delivered through interactive social media for health behaviour change, health outcomes, and health equity in the adult population. Cochrane Database Syst Rev. 2021;5(5):Cd012932.
- Hunter RF, de la Haye K, Murray JM, Badham J, Valente TW, Clarke M, et al. Social network interventions for health behaviours and outcomes: A systematic review and meta-analysis. PLOS Medicine. 2019;16(9):e1002890. doi: 10.1371/journal.pmed.1002890.

**Comment# 21. “As such, additional scientific inquiry and evidence is needed before any digital health can be recommended as a viable means to improve self-care in GDM.” Do you really mean “any” digital health intervention or just the ones mentioned in this paragraph. Please rephrase for clarity.**

We agree and thank the editor for pointing this out. We have now edited the paragraph to clarify this. The revised sentence now reads as follows:-

*Given the limited evidence base to support the overall use of digital health to improve self-care in GDM, additional scientific inquiry and evidence is needed before it can be recommended in routine medical practice.*

**Comment #22.. “This may stem from the lack of a standardised definition to report study compliance”. Can you provide the definitions used in the trials as a supplement table?**

We take note of the suggestion. We have now included the definitions used in the trials as suggested as S1 Table in the appendix.

**Comment #23 “We found that the use of social platforms and to a smaller extent mHealth had the most benefits, and thus should be considered the highest priority in future uptake.” Suggest deleting after the comma, too speculative.**
We thank the editor for the suggestion. We have now edited the sentence as suggested. The revised sentence now reads as follows:-

*We found that the use of social platforms and to a smaller extent mHealth had the most benefits.*

**Comment #24. “O’Brien in one of the earliest review on this topic examined how technology can be used to support lifestyle interventions in pregnant women.” Reference?**

We apologise for the oversight. We have now edited and included the reference for this statement.

- O'Brien CM, Cramp C, Dodd JM. Delivery of Dietary and Lifestyle Interventions in Pregnancy: is it Time to Promote the Use of Electronic and Mobile Health Technologies? Semin Reprod Med. 2016;34(02):e22-e7.

**Comment #25. “most of the results were similar in terms of maternal and neonatal outcomes”: “similar” is not a correct interpretation as previously mentioned.**

We take note of the editor’s concern. We have edited the sentence as suggested. The revised sentence now reads as follows:-

*The present meta-analysis showed some benefits of using digital health in improving glycaemic control and neonatal outcomes. As such, additional research is needed in view of their potential effectiveness.*

**Comment #26 S2 Table: Can you revise to make sure the comments are correct for all outcomes (e.g. caesarean)**

We take note and apologise for the oversight. We have now checked this for consistency.

**Comment #27. There are several typos throughout; suggest proof-reading.**

We have requested for a colleague to proof read this as suggested.

**Comment #28. Throughout the manuscript there are many instances where you should refer to “digital health interventions”, not just “digital health”.**

We take note of the suggestion. We have now edited this to ensure that these are referred to digital health interventions for consistency.

We wish to thank both reviewers and the editor for their time and effort in helping us to improve the clarity of our manuscript.

Yours truly

Shaun Lee

On behalf of the authors
