# Supplementary material for: Digital health interventions for gestational diabetes mellitus: A systematic review and meta-analysis of randomised controlled trials
Source: PLOS Digit Health. 2022 Feb 24;1(2):e0000015. doi: 10.1371/journal.pdig.0000015 (PMC9931335; doi:10.1371/journal.pdig.0000015)
Supplement: Attachment — Submitted filename: Revision letter.docx [file pdig.0000015.s006.docx]

14 December 2021

Liliana Laranjo
Section Editor
PLOS Digital Health

Dear Dr Liliana Laranjo

We are pleased to resubmit our revised manuscript entitled " Digital health interventions for gestational diabetes mellitus: A systematic review and meta-analysis of randomised controlled trials " to the PLOS Digital Health As suggested by the editor, we have revised our manuscript based upon the suggestions.

The editor had the following comments.

Comment #1. As mentioned in the previous comment, the authors can only say that the results were not significantly different, they cannot say they are similar.

We apologise for the oversight. We have now edited our manuscript to state that they were not significantly different. The revised abstract states as follows:-

Other maternal and foetal outcomes were not significantly different between both groups

The revised results now reads as follow:-

Delivery outcomes

However, there was no significant difference between both groups on reducing adverse outcomes such as pre-eclampsia/eclampsia or need for use of medication

Neonatal outcomes

Pooled analysis showed that the use of digital health interventions was not significantly different on all neonatal outcomes examined

In our revised discussion, it reads as follows:-

. In terms of adverse neonatal outcomes such as risk of hypoglycaemia and need for neonatal intensive care unit hospitalization results were not significantly different between both groups

In addition the Interpretation and future direction the discussion is also edited to state the following:-

Our results are timely as the incidence of GDM is increasing rapidly and with the explosion of use of digital health interventions currently. This is especially relevant with the growing interest in digital technologies and the increasing affordability of such technologies to date. However, as data are only derived from a very small cohort of subjects and the very long-term consequences on both maternal and foetal outcomes are currently lacking, further research is needed before digital health interventions can be used in clinical practice. As achieving optimal outcomes in GDM requires individuals to perform complex daily self-care tasks, it is important that any future intervention designed should adequately support and sustain these activities.[9] Indeed, in many of these trials, the human-computer interaction is often overlooked, due to poor study design which lacked a theoretical framework.

Comment #2. The wording of Comment #17 should be improved for clarity. “*The meta-analysis results suggest that intervention which involves the provision of education, monitoring and motivation by peers as well as healthcare providers were associated with improved glycaemic control. The combination of monitoring and education could have resulted in an increase the level of interaction between participants and healthcare providers and thus compliance to treatment advice.*

We take note of the suggestion. We have now revised our discussion paragraph to state the following:-

The meta-analysis results suggest that intervention which involves education and monitoring of mothers with GDM can lead to improved glycaemic control. This could possibly be due to these activities which increased the level of interaction between participants and healthcare providers and thus compliance to treatment advice.

We thank the editor for the opportunity to contribute to the journal and look forward to a favourable reply.

Yours truly

Shaun Lee

On behalf of the authors
